# Genome-wide analysis of transcriptome and histone modifications in *Brassica napus* hybrid

**DOI:** 10.3389/fpls.2023.1123729

**Published:** 2023-01-27

**Authors:** Meng Ma, Wenying Zhong, Qing Zhang, Li Deng, Jing Wen, Bin Yi, Jinxing Tu, Tingdong Fu, Lun Zhao, Jinxiong Shen

**Affiliations:** National Key Laboratory of Crop Genetic Improvement, Hubei Hongshan Laboratory, National Center of Rapeseed Improvement in Wuhan, Huazhong Agricultural University, Wuhan, China

**Keywords:** *Brassica napus*, heterosis, gene expression, epigenetic, histone modification

## Abstract

Although utilization of heterosis has largely improved the yield of many crops worldwide, the underlying molecular mechanism of heterosis, particularly for allopolyploids, remains unclear. Here, we compared epigenome and transcriptome data of an elite hybrid and its parental lines in three assessed tissues (seedling, flower bud, and silique) to explore their contribution to heterosis in allopolyploid *B. napus*. Transcriptome analysis illustrated that a small proportion of non-additive genes in the hybrid compared with its parents, as well as parental expression level dominance, might have a significant effect on heterosis. We identified histone modification (H3K4me3 and H3K27me3) variation between the parents and hybrid, most of which resulted from the differences between parents. H3K4me3 variations were positively correlated with gene expression differences among the hybrid and its parents. Furthermore, H3K4me3 and H3K27me3 were rather stable in hybridization and were mainly inherited additively in the *B. napus* hybrid. Together, our data revealed that transcriptome reprogramming and histone modification remodeling in the hybrid could serve as valuable resources for better understanding heterosis in allopolyploid crops.

## 1 Introduction

Heterosis, or hybrid vigor, is a fundamental phenomenon wherein hybrids have increased yield and biomass relative to their parents (Chen, 2013). This phenomenon has been widely used in crop breeding, such as in rapeseed (*Brassica napus*), rice (*Oryza sativa*), maize (*Zea mays*), sorghum (*Sorghum bicolor*), and tomato (*Solanum lycopersicum*) (Krieger et al., 2010; Li et al., 2015b; Shao et al., 2019; Li et al., 2020; Knoch et al., 2021). Although it has been extensively used in breeding for more than a century, the molecular mechanisms underlying heterosis remain largely unclear. Several classical genetic models, including dominance, over-dominance, and epistasis have been proposed. The dominance hypothesis involves the introduction of new favorable dominant alleles in hybrids (Jones, 1917; Birchler et al., 2010; Wu et al., 2021). The over-dominance hypothesis involves the superiority of heterozygous state compared to either parental homozygous state (Lariepe et al., 2012). The epistasis interaction hypothesis involves the role of ideal alleles in heterosis gain (Frascaroli et al., 2007). However, the exact molecular mechanisms of heterosis are not fully understood (Chen, 2010; Liu et al., 2020).

Epigenetic systems play important roles in regulating transcription and controlling diverse biological processes (He et al., 2011; You et al., 2017; Zeng et al., 2019; Zhao et al., 2020). Previous studies have illustrated that H3K4me3 is an active histone mark that is related to gene activation and accumulated predominantly in promoters of active genes in plants (Zhang et al., 2009; Zhang et al., 2015; Li et al., 2019). H3K27me3 is a classical transcriptionally repressive mark that is mainly located in the gene body region with high tissue specificity (Lafos et al., 2011; Wiles and Selker, 2017). Growing evidence has revealed that H3K27me3 is involved in developmental and environmental plant responses (e.g., salt stress, drought stress and heat stress) (Nishio et al., 2020; Yamaguchi, 2021).

Recent studies have indicated that epigenetic regulation is involved in heterotic phenotypes (Ni et al., 2009; He et al., 2010; Chen, 2013; Greaves et al., 2015; Yang et al., 2016; Ma et al., 2021). Epigenomic analyses have implied that the altered patterns of histone modifications (H3K4me2 and H3K9ac) are associated with expression levels of circadian clock genes that are related to energy production and storage in Arabidopsis hybrids (Ni et al., 2009). The altered histone modification patterns of some genes, such as FLC in Arabidopsis hybrid, could be correlated to heterotic traits (Zhu et al., 2017). Further, in Arabidopsis hybrids, the pattern of histone modification in parents is primarily inherited by the F1 hybrids, but some genes with non-additive gene expression also possess non-additive histone modification in Arabidopsis hybrids (Moghaddam et al., 2011; Dong et al., 2012). In addition, variation of H3K27me3 between parental lines may result in the allelic bias of H3K27me3 in Arabidopsis hybrids (Yang et al., 2016). Similarly, in rice and maize hybrids, the remodeling of histone modification was observed in some regions compared to the parents, which are associated with changes in gene expression (He et al., 2010; He et al., 2013a). Li et al. reported that histone modification plays an important role in non-additive gene expression in hybrid rice (Li et al., 2011). Studies of epigenetic mechanisms in heterosis have focused on the dicot model plant Arabidopsis and the monocot model plant rice; however, the epigenetic mechanisms of heterosis in allopolyploid plants remain poorly understood (Shen et al., 2017).

*Brassica napus* (AACC, 2n = 38, *B. napus*) is an allopolyploid crop that originated from interspecific hybridization between *Brassica rapa* (AA, 2n = 20) and *Brassica oleracea* (CC, 2n = 18) about 7500 years ago (Chalhoub et al., 2014). *B. napus* is cultivated as the second most-important edible oil crop in the world and also can be used as biofuels and bioplastics (Stahl et al., 2017). The yield and production of many *B. napus*-producing areas has increased due to the commercial use of F1 hybrids (Wang et al., 2021a). A recent work indicated that the Cn subgenome has a larger influence on heterosis than the An subgenome in *B. napus* (Wang et al., 2021a). DNA methylation and siRNA were also involved in heterosis through regulating gene expression in *B. napus* hybrid (Shen et al., 2017). However, comprehensively comparative epigenomic and transcriptomic analyses in different tissues of *B. napus* hybrid remain largely unexplored.

2063A (an excellent *pol* CMS line) and B409 (restorer line) are the parents of the elite hybrid Huayouza 62 (HZ62), which exhibits superiority in a large array of agronomic traits including yield, biomass, and adaptability. HZ62 used to be one of the most cultivated varieties in China and has multi-functions, such as soil, vegetable, flower, forage, and fertilizer. Our previous study profiled high-quality epigenome maps of the two parents and revealed that the biased transcription between the An and Cn subgenomes is tightly associated with the asymmetric epigenomic (Zhang et al., 2021). Here, we generated high-quality H3K4me3, H3K27me3, and transcriptome data for three assessed tissues (seedling, flower bud, and silique) of HZ62 to better understand *B. napus* heterosis. We identified genome-wide transcriptional and histone modification (H3K4me3, H3K27me3) variations between HZ62 and its two parents and found a relationship between these two types of polymorphisms. Collectively, our results suggested that epigenetic and gene expression variation occurring in hybrids may play a vital role in heterosis.

## 2 Materials and methods

### 2.1 Plant materials and growth conditions

**Figure 1:**
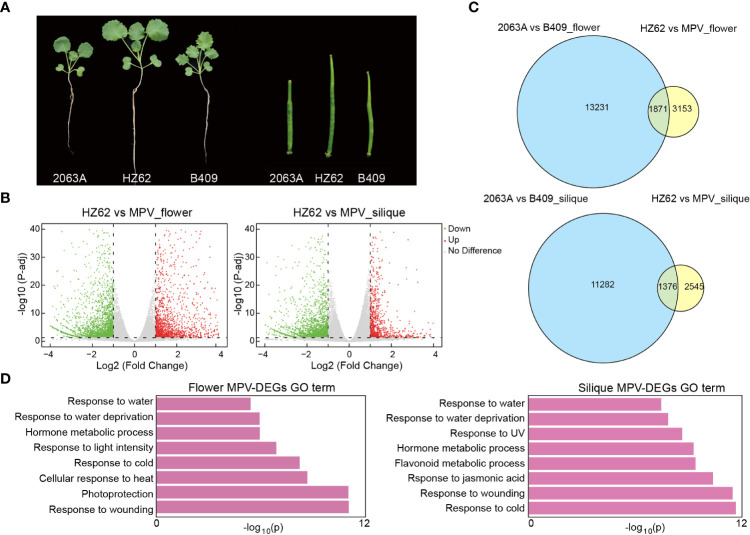
Differentially expressed genes (DEGs) in hybrid compared with MPVs (mid-parent values). **(A)** Phenotypic difference and heterosis in the seedling (2-week-old plants) and silique (10 days after pollination) tissues of hybrid. **(B)** Volcano plot of MPV-DEGs in flower bud and silique tissues of hybrid [false discovery rate <0.05, log2 (fold change) > 1 or < –1]. **(C)** Overlap of DEGs between parents and MPV-DEGs in flower bud. **(D)** GO terms were associated with MPV-DEGs in the indicated tissues.

*Brassica napus* F1 hybrid HZ62 and its parents 2063A (male-sterile line) and B409 (restorer line) were used in this study (**Figure 1A**; **Supplementary Figure S1**). For seedlings, germinated seeds were grown in a greenhouse with a day/night cycle of 16/8h and a temperature of 22°C/18°C for 2 weeks (Wan et al., 2017). For flower buds (≤2 mm) and siliques (10 days after pollination), materials were grown in experimental farm under natural conditions at Huazhong Agricultural University in Wuhan, China.

### 2.2 RNA-seq library preparation

The RNA-seq data of the parents used in this study were from a previous study (Zhang et al., 2021). Total RNA was isolated from the tissues (seedling, flower bud, silique) using the RNeasy Plant Mini Kit (QIAGEN, 74904) according to the manufacturer’s instructions. About 2 µg of RNA was used for library construction using an Illumina TruSeq RNA Kit according to the manufacturer’s instructions. RNA sequencing was performed using the Illumina Hiseq X Ten system. RNA-seq data were obtained for two biological replicates.

### 2.3 ChIP-seq library preparation

ChIP-seq (chromatin immunoprecipitation followed by sequencing) libraries of HZ62 were prepared as previously described (Zhao et al., 2020; Zhang et al., 2021). Briefly, tissues were collected and cross-linked using 1% formaldehyde (Sigma, F8775) and quenched with 0.2 M glycine (Sigma, G7126) at room temperature. Then, 1–1.5 g of the samples was used for each library. After grinding into a fine powder with liquid nitrogen, cell lysis was performed by 1% SDS (Sigma, AM9822) at 4°C. Then, the chromatin was fragmented into 200–600 bp segments by sonication using a Bioruptor (Diagenode). ChIP was performed using H3K4me3 and H3K27me3 antibodies (Abclonal, A2357 for H3K4me3, and A2363 for H3K27me3). Then, the protein-DNA complexes were reverse-cross-linked using proteinase K (Invitrogen, AM2546) at 55°C overnight. ChIP-DNA was extracted using phenol:chloroform:isoamyl alcohol (Sigma, P3803). ChIP-DNA libraries were constructed by using NEBNext Ultra II DNA library prep kit for Illumina (New England BioLabs, E7645) according to the manufacturer’s guidelines. Finally, the DNA libraries were sequenced with the Illumina HiSeq X Ten system. ChIP-seq data were obtained for two biological replicates.

### 2.4 RNA-seq analysis

Adaptors and low-quality reads were first trimmed using Fastp with default parameters (Chen et al., 2018). Clean reads were mapped to the *Brassica napus* ZS11 genome using Hisat2 with default parameters (Kim et al., 2019; Chen et al., 2021), and then SAMtools with parameters “-q 30 -f 2” was used to remove the low mapping quality reads (Li et al., 2009). GATK MarkDuplicates was used to remove PCR-duplicated reads. BAM files were then normalized and converted to BigWig format using the Deeptools bamCoverage function with parameters “–normalizeUsing RPKM –binSize 10” to configure the tracks in IGV (DePristo et al., 2011; Thorvaldsdóttir et al., 2013; Ramírez et al., 2016). Next, reads numbers per gene were counted using FeatureCounts (Liao et al., 2014). The trimmed mean of M-values (TMM) method of EdgeR was used to normalize the read counts per gene, then the mid-parent value (MPV) for each tissue was designated as the mean of the parental TMM (Robinson et al., 2010). EdgeR was used to perform a differential expression test between HZ62 and MPV, using the threshold false discovery rate (FDR) < 0.05 && log2 (fold change) > 1 or < −1 (Robinson et al., 2010).

**Figure 2:**
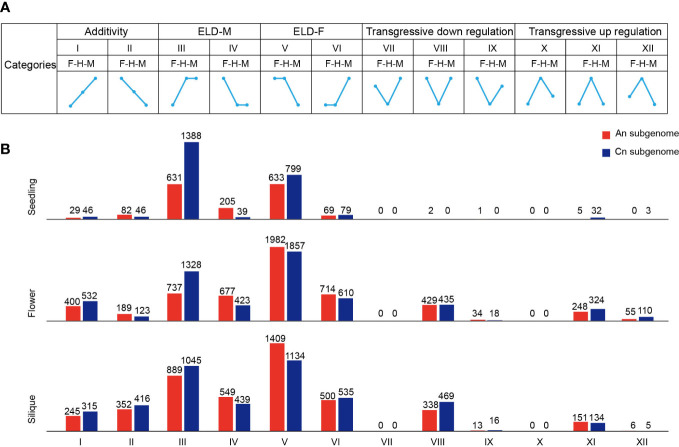
Classification of expressed genes in F1 hybrid. **(A)** Gene expression patterns of 12 groups in hybrid. F, female parent; H, hybrid; M, male parent. ELD, expression level dominance. **(B)** Number of genes in 12 groups in the seedling, flower bud, and silique.

As for the gene expression pattern analysis, 12 different expression patterns were classified using published methods (Yoo et al., 2013). Panels I–XII (see **Figure 2A**) indicated 12 possible expression classes between the parents and hybrid, with the roman numerals indicating the same classes used by Yoo et al. Thus, for example, panel I shows genes for which the male parent was differentially expressed and downregulated relative to the hybrid; meanwhile, the hybrid was differentially expressed and downregulated relative to the female parent, and there was no significant difference between the hybrid and MPV at the same time. GO and Kyoto Encyclopedia of Genes and Genomes (KEGG) enrichment analyses were performed using the R package of ClusterProfiler (Yu et al., 2012), and the significant threshold q value was <0.05.

### 2.5 ChIP-Seq analysis

Fastp with default parameters was used to trim adaptors and low-quality reads in raw reads (Chen et al., 2018). Clean reads were mapped to the *B. napus* “ZS11” (Zhongshuang 11) genome by BWA-MEM with default parameters (Li and Durbin, 2009; Chen et al., 2021). SAMtools was used to remove the low mapping quality reads with parameters “-q 30 -f 2” (Li et al., 2009), and GATK MarkDuplicates was used to remove duplicated reads (DePristo et al., 2011). Deeptools bamCoverage with parameters “–normalizeUsing CPM -e –binSize 10” was used to normalize and convert the last BAM file which was generated by the above processing steps to BigWig format (Ramírez et al., 2016). Next, ChIP-seq narrow peaks were called by MACS2, with parameters “-f BAMPE -B -g 921913538 -q 1e-5” (Liu, 2014). A heatmap and peak profile was generated by the Deeptools ComputeMatrix and PlotHeatmap commands (Ramírez et al., 2016). As for the differential peak analysis, first, the peak regions of two different groups were combined using the Bedtools merge command (Quinlan and Hall, 2010). Then, each sample’s read counts in the merged peak regions were counted by Bedtools intersect with parameter “-c” (Quinlan and Hall, 2010). Lastly, the differential peaks test was performed with DESeq2, with a threshold FDR of < 0.05 && log2 (fold change) > 1 or < − 1 (Love et al., 2014). GO enrichment analyses were performed using the R package of ClusterProfiler (Yu et al., 2012).

### 2.6 Quantitative real-time PCR

Total RNA was isolated from the tissues (seedling, flower bud, silique) using the RNeasy Plant Mini Kit (QIAGEN, 74904) according to the manufacturer’s instructions for conducting PCR. Six genes relevant to starch metabolism were selected to validate the RNA-seq data *via* qRT-PCR. Primers were designed using Primer 5.0 and listed in **Supplementary Table S5**. cDNA was synthesized by ABScript III RT Master Mix (Abclonal, RK20429). qRT-PCR was performed using the 2X Universal SYBR Green Fast qPCR Mix (Abclonal, RK21203). The reactions were performed using a CFX96 real-time system (Bio-Rad). The housekeeping gene *UBC10* served as an internal control. All qRT-PCR reactions included three biological replicates, and each biological replicate had three technical replicates.

## 3 Results

### 3.1 A small fraction of expressed genes show non-additive expression in the hybrid

Phenotypically, HZ62 has significantly increased biomass and silique length (*t*-test, *p* < 0.001) (**Figure 1A**; **Supplementary Figure S2**). To investigate the dynamic changes of gene expression between the elite hybrid *B. napus* HZ62 and its parents, we performed RNA-seq in the seedling, flower bud, and silique tissues. The RNA-seq data of the parents used in this study were published previously (Zhang et al., 2021). More than 93% of the sequenced reads were mapped to the reference *B. napus* genome (**Supplementary Table S1**). The Pearson’s correlation coefficient between biological replicates for each tissue was high (0.94–0.99) (**Supplementary Figure S3**; **Supplementary Table S1**). Genes in hybrids that had significantly different expression levels compared to the MPV were identified as MPV-differentially expressed genes (MPV-DEGs) or non-additive expression genes. The significance threshold was set as FDR <0.05 and with log2 (fold change) > 1 or < – 1. Totals of 109 (0.15% of total expressed genes), 5003 (6.9% of total expressed genes), and 3914 MPV-DEGs (5.4% of total expressed genes) were detected in the seedling, flower bud, and silique tissues, respectively (**Figure 1B**; **Supplementary Figure S4**), which indicated that only a small fraction of total expressed genes (0.15%–6.9%) showed non-additive expression, and most of them were additively expressed in the hybrid. Furthermore, the number of MPV-DEGs distributed across the An and Cn subgenomes was similar (**Supplementary Figure S4**).

Interestingly, we obtained few common and mostly unique MPV-DEGs in the seedling, flower bud, and silique tissues (**Supplementary Figure S5A**). For example, only 101 (~5.4%) upregulated MPV-DEGs in flower bud overlapped with those in other tissues (**Supplementary Figure S5A**). The results indicated that different genes were reprogrammed in the hybrid at different developmental stages. Among the MPV-DEGs, about 36% overlapped with parental DEGs, and more downregulated MPV-DEGs overlapped with parental DEGs in the seedling and silique; these differences were significant (χ^2^ test, *p* < 0.001) (**Figure 1C**; **Supplementary Figures S5B, C**). These results indicated that many non-additive expression genes in the hybrid exhibited an expression difference between parents. To examine the functions of MPV-DEGs, gene ontology (GO) functional enrichment analysis was performed. MPV-DEGs were significantly enriched in the stress response and hormone response pathways in flower bud and silique (**Figure 1D**). In the seedling, MPV-DEGs were significantly enriched in glucosinolate catabolic process and stress response pathways (**Supplementary Figure S5D**). These results suggested that non-additively expressed genes might promote stress tolerance of hybrid. A KEGG enrichment analysis of MPV-DEGs showed that they were mainly enriched in circadian rhythm and plant hormone signal transduction pathways in flower bud and silique (**Supplementary Figure S6**). Taken together, these results suggested that the hybridization of two parental inbred lines of *B. napus* can lead to a small number of non-additively expressed genes that are mainly related to the stress response pathway.

### 3.2 Parental expression level dominance genes are overrepresented in the hybrid

Expression level dominance (ELD) is a phenomenon in which hybrids exhibit expression patterns similar to those of one of their parents (Yoo et al., 2013). To investigate gene expression patterns in the hybrid, we classified genes into 12 categories based on previous studies, including additivity, parental ELD, and transgressive expression (**Figure 2A**) (Yoo et al., 2013). A great number of parental ELD genes were identified in all three tissue types (**Figure 2B**). For instance, 2019 genes in the seedling tissue of the hybrid showed a high male dominance expression level. In contrast, a limited number of genes exhibited additivity and transgressive expression in these tissues (**Figure 2B**). In the seedling, ELD genes had an asymmetric distribution across the An and Cn subgenomes, but were not observed in flower bud or silique (**Figure 2B**). Moreover, the proportion of high-parental ELD genes was significantly higher than low-parental ELD genes in the seedling, flower bud, and silique (Z-test, *p* < 0.001) (**Figure 2B**). In the hybrid, most ELD genes displayed a significantly higher portion paternal dominant expression in the seedling, whereas maternal dominant expression was significantly higher in flower bud and silique (Z-test, *p* < 0.001) (**Figure 2B**).

To explore the biological functions of ELD genes in the hybrid, we performed a GO enrichment analysis. In the seedling, the ELD genes were significantly enriched in ATP synthesis/metabolic process, circadian rhythm, and stress to response pathways (**Supplementary Figure S7**). Among the ELD genes, many were previously reported to be associated with important traits or heterosis, such as flowering and grain yield, including *TEMPRANILLO 1* (*TEM1*, *ZS11C05G023770*), *Empfindlicher im Dunkelroten Licht 1* (*EID1*, *ZS11C09G001910*), *LATE ELONGATED HYPOCOTYL* (*LHY*, *ZS11A10G000850*), and succinate dehydrogenase (*SDH2*, *ZS11A02G037800*) (Marrocco et al., 2006; Fujiwara et al., 2008; Hu et al., 2021; Li et al., 2021). In addition, the GO enrichment analysis revealed that the ELD genes in the flower bud were relatively enriched in processes related to flower development, indicating that ELD genes play a key role in flower development (**Supplementary Figure S7**). Taken together, our results indicated that ELD genes involved in important biological processes, such as ATP synthesis/metabolic and circadian rhythm, were overrepresented in the hybrid and may play a critical role in heterosis in *B. napus*.

### 3.3 Epigenetic variations among parental lines and hybrid

**Figure 3:**
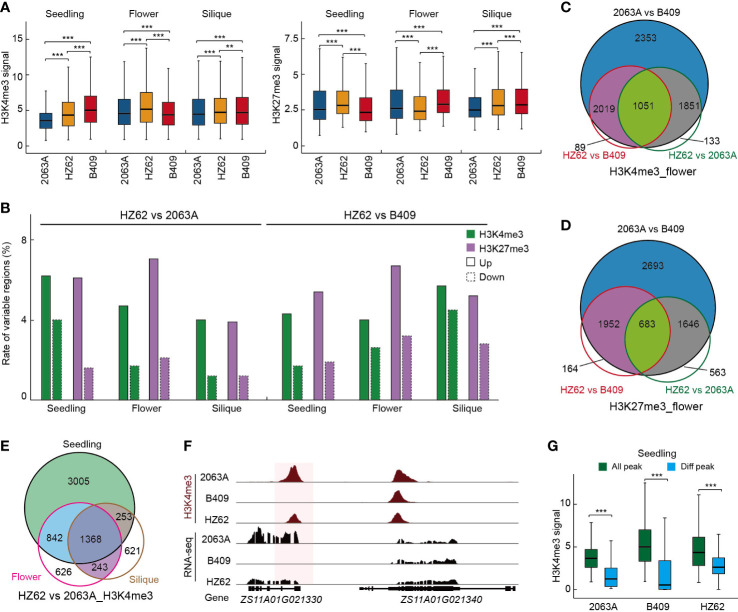
Epigenetic variations among hybrid and parents. **(A)** Peak intensities of histone modifications in hybrid and parents in each tissue. ***p < 0.001 from Wilcoxon test. **(B)** The rate of variable H3K4me3 and H3K27me3 among hybrid and parents in the indicated tissues. **(C)** Overlap of H3K4me3 variation among parents and hybrid. **(D)** Overlap of H3K27me3 variation among parents and hybrid. **(E)** Overlap of H3K4me3 differences between hybrid and parents among three assessed tissues. **(F)** Example of differential histone modification regions in parental lines and hybrid. **(G)** H3K4me3 intensities of all H3K4me3 loci and variable H3K4me3 loci in the indicated varieties. ***p < 0.001 from Wilcoxon test. Asterisks indicate statistically significant differences.

To explore the epigenetic variations between the parents and the hybrid, we employed ChIP-seq to generate the genome-wide H3K4me3 and H3K27me3 profiles of HZ62 for the three assessed tissues. The ChIP-seq data generated in this study were of high quality and reliable (**Supplementary Table S2**; **Supplementary Figure S8**). For each sample, two biological replicates were conducted, and the inter-replicate correlation was high for the ChIP-seq assay (R > 0.95, **Supplementary Table S2**; **Supplementary Figure S8**). The ChIP-seq datasets of the parental lines used in this study were published previously (Zhang et al., 2021). Box plots of the histone modification signals clearly revealed that H3K4me3 and H3K27me3 showed significant differences between the parents and hybrid for the three assessed tissues (Wilcoxon test, ***p < 0.001) (**Figure 3A**). In the seedling, the hybrid exhibited a significantly higher peak intensity of H3K4me3 than maternal parent 2063A, whereas it was significantly lower than paternal parent B409 (**Figure 3A**). In the flower bud and silique, H3K4me3 signals were significantly increased in the hybrid compared to the parents (**Figure 3A**). The levels of H3K27me3 were increased in the seedling and decreased in the flower bud in the hybrid (**Figure 3A**). However, H3K27me3 signals in the hybrid were significantly higher than 2063A but lower than B409 in the silique (**Figure 3A**). In the hybrid, more upregulated H3K4me3 and H3K27me3 loci were observed in the seedling, flower, bud and silique, and the differences were significant (χ^2^ test, p < 0.001) (**Figure 3B**, **Supplementary Table S3**). In addition, ~5% and ~5.3% of upregulated H3K4me3 and H3K27me3 loci were observed in HZ62 *vs.* 2063A and HZ62 *vs.* B409, respectively (**Figure 3B**). In contrast, ~1.9% and ~ 3% of downregulated H3K4me3 and H3K27me3 loci were observed in HZ62 *vs.* 2063A and HZ62 *vs.* B409, respectively (**Figure 3B**). Taken together, our results indicated significant differences of histone mark levels in the hybrid compared to its parental lines, which displayed a differential tendency in different tissues.

Differentially histone-modified regions between 2063A and B409 were further investigated. In the seedling, flower bud, and silique, 8297, 7274, and 9261 differentially H3K4me3-modified loci (15%–19% of the total H3K4me3 loci); and 8566, 6974, and 6726 differentially H3K27me3-modified loci (23%–27% of the total H3K27me3 loci), were identified between 2063A and B409 (FDR < 0.05, log2 fold change > 1 or < –1), respectively (**Supplementary Table S3**). We found more dynamic changes in the repressive mark H3K27me3 than in the active histone mark H3K4me3 between parental lines (χ^2^ test, *p* < 0.001) (**Supplementary Table S3**). Notably, 80%–95% of differentially H3K4me3-modified regions and 85%–91% of differentially H3K27me3-modified regions in the hybrid overlapped with the differentially histone-modified regions between parents for the seedling, flower bud, and silique (**Figures 3C, D**; **Supplementary Figures S9A, B**). This result suggested that regions with histone modification differences between the parental lines predominantly exhibited differential modification in the hybrid compared with the parents (**Figures 3C, D, F**; **Supplementary Figures S9A, B**). Both H3K4me3- and H3K27me3-conserved regions in the parental lines and hybrid displayed significantly higher peak intensities, whereas the differentially histone-modified regions exhibited significantly lower peak intensities in the seedling, flower bud, and silique (Wilcoxon test, ***p < 0.001) (**Figure 3G**; **Supplementary Figure S10**). Furthermore, we observed that the number of differentially histone-modified regions that overlapped among the tissues (25%–55% and 23%–51% for H3K4me3 and H3K27me3, respectively) was significantly higher than that of MPV-DEGs in the hybrid (χ^2^ test, *p* < 0.001) (**Figure 3E**; **Supplementary Figures 9C, D**; **Supplementary Figure 5A**), which indicated more differences in gene expression dynamics than histone modification variations during seedling to silique development.

### 3.4 Epigenomic variations correlated with gene expression variations among parental lines and hybrid

**Figure 4:**
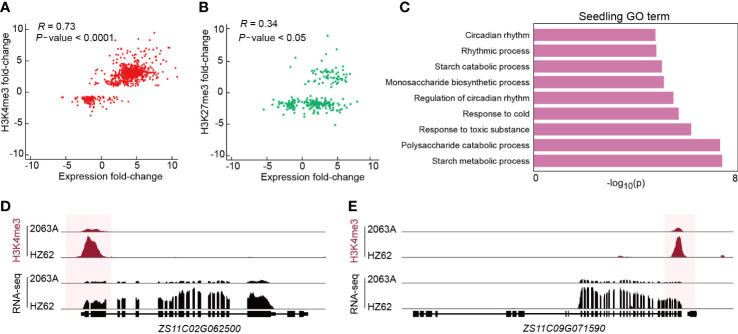
**(A, B)** Correlations between differential histone modification (FDR < 0.05, log2 (fold change) > 1 or < –1) and gene expression (FDR < 0.05, log2 (fold change) > 1 or < –1). The statistical analysis wad performed using the t-test. **(C)** Enriched GO terms of genes associated with differentially H3K4me3-modified regions between HZ62 and 2063A in seedling. **(D, E)** Genome browsers of differentially H3K4me3-modified regions and expressed genes in HZ62 and 2063A.

To verify the relationship between epigenetic variations and transcriptional divergence in the hybrid, we calculated the correlation coefficient between differential gene expression and histone modification. The intensities of H3K4me3 had a strong positive correlation with the changes of gene expression between parent and hybrid in all three assessed tissues (Pearson correlation = 0.63–0.83) (**Figure 4A**; **Supplementary Figure S9**). In contrast, the intensities of H3K27me3 displayed a weak positive correlation with gene expression changes between parent and hybrid in all three assessed tissues (Pearson correlation = 0.05–0.51) (**Figure 4B**; **Supplementary Figure S11**). Based on the GO enrichment analysis, genes with differential H3K4me3 between 2063A and HZ62 in the seedling were significantly enriched in the circadian rhythm, starch metabolism, and stress to response processes (**Figure 4C**), which have been reported to be associated with growth vigor in *Arabidopsis* hybrids (Ni et al., 2009). In the seedling, both glucan-water dikinase (*GWD*, *ZS11C02G062500*) and pullulanase/limit dextrinase (*LDA*, *ZS11C09G072500*) are involved in starch metabolism (Smith et al., 2005; Ni et al., 2009; Wang et al., 2021b). *GWD* and *LDA* were upregulated in *B. napus* hybrid, and their changes in expression were correlated with changes in H3K4me3 (**Figures 4D, E**). In the flower bud, upregulated genes associated with jasmonic acid, including acyl-activating enzyme gene (*AEE*, *ZS11C02G067470*), *ALLENE OXIDE CYCLASE 3* (*AOC3*, *ZS11C09G031080*), with higher level of H3K4me3 (**Supplementary Figure S12**). The gene involved in photosynthesis *LIGHT-HARVESTING CHLOROPHYLL B-BINDING 2* (*LHCB2, ZS11C02G057950*) was upregulated in silique, and was associated with higher level of H3K4me3 (**Supplementary Figure S12**). We selected six genes involved in starch metabolism including *GWD* (*ZS11A09G059080, ZS11C08G025260*, *ZS11A09G005180, ZS11C02G062500*, *ZS11C09G005880*) and *LDA* (*ZS11C09G072500*) for quantitative analysis; the quantitative results were consistent with the results of the transcriptome data (**Supplementary Figure S13**). Genes associated with starch metabolism, jasmonic acid metabolism, and photosynthesis pathways had increased gene expression in hybrid and may be related with greater growth and defense response of the hybrid. Collectively, our results suggested that H3K4me3 variations are correlated with gene expression differences in the hybrid following hybridization, which are associated with heterosis in *B. napus*.

### 3.5 Additive inheritance of epigenetic modification in F1 hybrid

**Figure 5:**
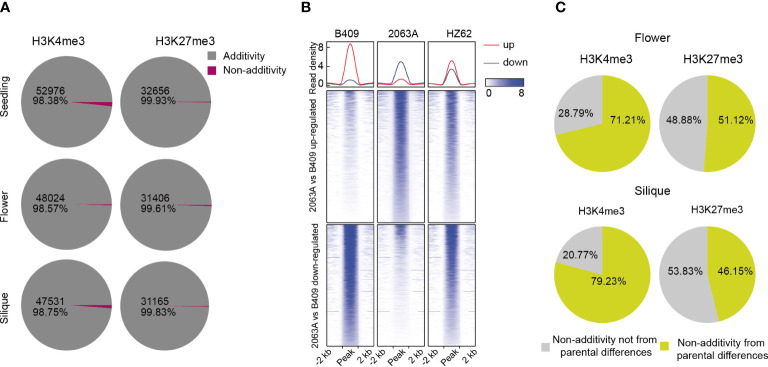
Overview of differentially histone-modified regions in hybrid compared with MPV. **(A)** Numbers of differentially histone-modified regions in the hybrid compared with MPV in seedling, flower bud and silique [FDR <0.05, log2 (fold change) > 1 or < -1]. **(B)** Comparison of intensities of H3K4me3 in differentially histone-modified regions among parents and hybrid. **(C)** Percentage of non-additively modified regions in hybrid originating from parental differentially histone-modified regions.

To better understand the variations of epigenomic modifications following hybridization in *B. napus*, we analyzed patterns of histone modifications in the hybrid compared with its parents for the three assessed tissue types. H3K4me3-modified and H3K27me3-modified regions in the hybrid that had significantly differential modification levels compared to the expected MPV (mean modification level of the two parental values) (FDR <0.05, log2 fold change > 1 or < –1) were identified as non-additively modified region; otherwise, they were identified as additively modified regions. Interestingly, almost all H3K4me3- and H3K27me3-modified regions displayed an additive nature in the hybrid for all three tissues (**Figure 5A**). The active mark H3K4me3 possessed a smaller proportion of additively modified regions than the repressive mark H3K27me3 (98.38%–98.75% and 99.61%–99.93% for H3K4me3 and H3K27me3, respectively) (**Figure 5A**), and these differences were significant in the seedling and silique (χ^2^ test, *p* < 0.001). We observed that it was more of downregulation than upregulation (compared with MPV) of H3K4me3 in the hybrid, and the difference were significant in the silique (Z-test, *p* < 0.001) (**Supplementary Table S4**). In contrast, significantly more upregulation-modified regions (compared with MPV) of H3K27me3 were obtained in the hybrid (Z-test, *p* < 0.01) (**Supplementary Table S4**). Further, it was shown that the hybrid exhibited intermediate patterns of H3K4me3 and H3K27me3 that lay between its parental extremes (**Figure 5B**; **Supplementary Figures S14, 15**).

We then explored the non-additively histone-modified regions in the hybrid, where substantial numbers of such regions also showed differential modification between parental lines (**Figure 5C**; **Supplementary Figure S12**). About 54.3%–79% of H3K4me3-modified regions, showing non-additive inheritance in the hybrid, were derived from the differential modification between parents (54.3%, 71%, and 79%, seedling, flower bud, and silique, respectively) (**Figure 5C**; **Supplementary Figure S16**). As for H3K27me3-modified regions, approximately 14%–51% of non-additively modified regions were originated from the differential modification between parental lines (14%, 51% and 46% for seedling, flower bud, and silique, respectively), suggesting that epigenome divergence between parents needs to be remodeled during hybridization (**Figure 5C**; **Supplementary Figure S12**). Taken together, H3K4me3 and H3K27me3 are primarily an additive inheritance in the *B. napus* hybrid.

## 4 Discussion

Heterosis has been widely used in crop breeding to improve agricultural productivity (Schnable and Springer, 2013; Xiao et al., 2021; Liu et al., 2022). Accumulating evidence shows that transcriptome reprogramming and epigenome remodeling occur in the genome of hybrids compared with their parents (Groszmann et al., 2011; Greaves et al., 2015; Botet and Keurentjes, 2020; Sinha et al., 2020; Wang and Wang, 2022). In diploid plants, such as *Arabidopsis thaliana*, rice, and maize, research on the mechanism of transcriptome and epigenetic variation in hybrids has made some relevant progress (Ni et al., 2009; He et al., 2010; Shen et al., 2012; He et al., 2013a; Zhu et al., 2017; Wang and Wang, 2022). However, there are few studies on epigenetic variation in polyploid hybrids and its relationship with heterosis. *B. napus* is a typical allopolyploid that contains two sets of subgenomes. The hybrids of *B. napus* are widely used in agricultural production and have made significant economic impact (Chalhoub et al., 2014; Wang et al., 2018; Lu et al., 2019). In this study, we integrated transcriptome data and histone modifications (H3K4me3 and H3K27me3) for two *B. napus* inbred lines and their hybrid in three tissue types (seedling, flower, bud and silique) to investigate their roles in the establishment of heterosis.

Non-additive gene expression has been considered a specific expression model in hybrids that which could potentially be considered the cause of generating heterotic phenotypes (Fujimoto et al., 2012; Groszmann et al., 2014; Li et al., 2015a; Zhu et al., 2016; Yang et al., 2017; Zhao et al., 2019). We identified a number of non-additive expression genes, approximately 0.15%–6.9% of total expressed genes, that showed non-additive expression in the F1 hybrid. Based on a GO analysis, non-additive genes were significantly enriched in the stress to response pathway (**Figure 1**; **Supplementary Figure S5D**). Previously, transcriptome analyses between parents and hybrids reported only 2.8% non-additively expressed genes in hybrid rice, 10% in hybrid maize, and 0.8%–2.3% in *B. napus* (Song et al., 2010; Paschold et al., 2012; Shen et al., 2017). Collectively, these findings suggested that heterosis may occur due to the aggregation of important genes in parents into non-additive expression patterns in hybrids.

Parental ELD widely exists in polyploid hybrids and has been observed in cotton, rapeseed, and wheat, and ELD genes in hybrids are related to important biological functions (Yoo et al., 2013; Li et al., 2014; Yoo et al., 2014; Shen et al., 2017; Shahzad et al., 2020). In this study, we determined that a large number of ELD genes, especially high-parental ELD genes, were overrepresented in three tissue types of the hybrid (**Figure 2**). ELD genes were enriched in ATP metabolic, stress response, and circadian rhythm processes in the hybrid (**Supplementary Figure S7**), which have been published to enhance growth of plants (Ni et al., 2009; Miller et al., 2015; Murata and Nishiyama, 2018). This would imply that polyploid hybrids integrate the ideal genes of parental inbred lines, thus showing superior performance than their parents.

Recent studies have demonstrated that epigenetic modification in hybrids is associated with the potential molecular mechanism of hybrid vigor (Chen, 2013; He et al., 2013b; Offermann and Peterhansel, 2014; Dapp et al., 2015). Our comprehensive analysis showed significant differences in histone mark levels (H3K4me3, H3K27me3) between the parents and the hybrid (**Figure 3A**). The upregulation of H3K4me3 and H3K27me3 loci occurred more often than downregulation in the hybrid compared with its parents (**Figure 3B**). Notably, we found that most of the regions with histone modification variations in the hybrid originated from the regions with histone modification differences between parents (**Figures 3C, D, F**; **Supplementary Figures S9A, B**). The differential histone modification regions primarily showed lower peak intensities compared with the total histone modification regions (**Figure 3G**; **Supplementary Figure S10**). Furthermore, we found that there was a significantly positive correlation between changes of active mark H3K4me3 in active promoter regions and variation of gene expression in the hybrid, and the genes related to the changes of H3K4me3 were mainly enriched in stress response, biological rhythm, and starch synthesis/metabolism pathways (**Figure 4**). For instance, *GWD* gene expression was increased and with a higher level of H3K4me3 in the seedling tissue of the hybrid compared with its parents (**Figure 4D**). *GWD* is a vital enzyme that plays an important role in starch metabolism in source tissues (Skeffington et al., 2014; Zhou et al., 2017). In rice, the increase of *GWD* gene expression displayed improvements in many key traits, including yield, quality, grain shape, stress tolerance, and seed germination (Wang et al., 2021b). Together, these results indicated that the epigenetic remolding that occurred during *B. napus* hybridization was related to transcriptional reprograming, and both play important roles in the establishment of heterosis in *B. napus*.

Genetic differences are the basis of phenotypic differences between hybrids and parents (Badiane et al., 2012; Schnable and Springer, 2013; Boeven et al., 2020). Previous studies described that gene expression in hybrids will be affected by genetic differences, and the degree of heterosis is correlated with the genetic differences between the parental lines (He et al., 2010; Jaikishan et al., 2010; Paschold et al., 2012; Hao et al., 2015). Meanwhile, our results suggested that epigenetic differences in parental lines led to epigenetic differences between hybrid and parents, and epigenetic variations were correlated with gene expression. Many recent studies also support the idea that epigenetic difference is involved in heterosis (He et al., 2010; Chen, 2013; Groszmann et al., 2013). In addition, work in *Arabidopsis* has shown that epigenetic differences between parents can directly or indirectly influence heterosis in hybrids, independent of genetic differences (Lauss et al., 2018). Together, these observations may illustrate that genetic and epigenetic differences between parental inbred lines can act cooperatively or independently in heterosis.

In summary, the results of our study illustrate the dynamics of histone modifications and transcriptomes during *B. napus* hybridization in three pivotal developmental stages. We reveal the correlation of gene expression and histone modification variations among hybrid and parental lines, indicating that their variations are associated with heterosis establishment in the *B. napus* hybrid. However, we only explored two histone marks in this study. Therefore, more epigenetic marks need to be studied to uncover the epigenetic mechanisms of heterosis.

## Data availability statement

The datasets presented in this study can be found in online repositories. The names of the repository/repositories and accession number(s) can be found in the article/**Supplementary Material**.

## Author contributions

JS and LZ conceived the project and designed experiments. MM generated the datasets with the assistance of QZ. WZ performed data analysis. MM and WZ interpreted data and wrote the manuscript. LD, JW, BY, JT, and TF provided suggestions for the design of the study. All authors contributed to the article and approved the submitted version.
