# Corrigendum: Genome-wide analysis of transcriptome and histone modifications in *Brassica napus* hybrid

**DOI:** 10.3389/fpls.2023.1232986

**Published:** 2023-07-13

**Authors:** Meng Ma, Wenying Zhong, Qing Zhang, Li Deng, Jing Wen, Bin Yi, Jinxing Tu, Tingdong Fu, Lun Zhao, Jinxiong Shen

**Affiliations:** National Key Laboratory of Crop Genetic Improvement, Hubei Hongshan Laboratory, National Center of Rapeseed Improvement in Wuhan, Huazhong Agricultural University, Wuhan, China

**Keywords:** *Brassica napus*, heterosis, gene expression, epigenetic, histone modification

In the published article, there was an error in the **Funding** statement, page nine.

The previous Funding statement:

“This research was funded by the National Key Research and Development Program of China (202022YFD1200804), and the Program for Modern Agricultural Industrial Technology System (CARS-12).”

The corrected Funding statement appears below:

“This research was funded by the National Key Research and Development Program of China (2022YFD1200804), and the Program for Modern Agricultural Industrial Technology System (CARS-12).”

The authors apologize for this error and state that this does not change the scientific conclusions of the article in any way. The original article has been updated.

