# Supplementary material for: Genome-wide analysis of transcriptome and histone modifications in Brassica napus hybrid
Source: Front Plant Sci. 2023 Jan 27;14:1123729. doi: 10.3389/fpls.2023.1123729 (PMC9911877; doi:10.3389/fpls.2023.1123729)
Supplement: Supplementary file 1 [file DataSheet_1.pdf]

Supplementary Material

Genome-wide analysis of transcriptome and histone modifications in *Brassica napus* hybrid

Meng Ma†, Wenying Zhong†, Qing Zhang, Li Deng, Jing Wen, Bin Yi, Jinxing Tu, Tingdong Fu, Lun Zhao and Jinxiong Shen *

***Correspondence:**
Jinxiong Shen
jxshen@mail.hzau.edu.cn

Supplementary Figures

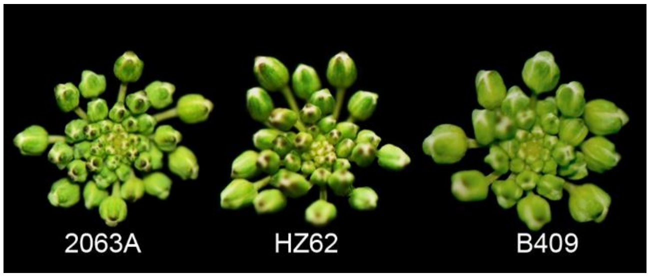

Supplementary Figure 1. Flower bud morphology in parents and their hybrid.

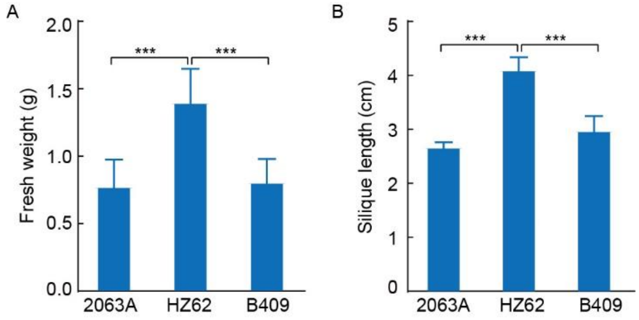

Supplementary Figure 2. Heterosis of hybrid in seedling and silique. **(A)** Comparison of fresh weight among parents and their hybrid. **(B)** Comparison of silique length among parents and their hybrid. The statistical analysis was performed using the t-test. ***$p < 0.001$.

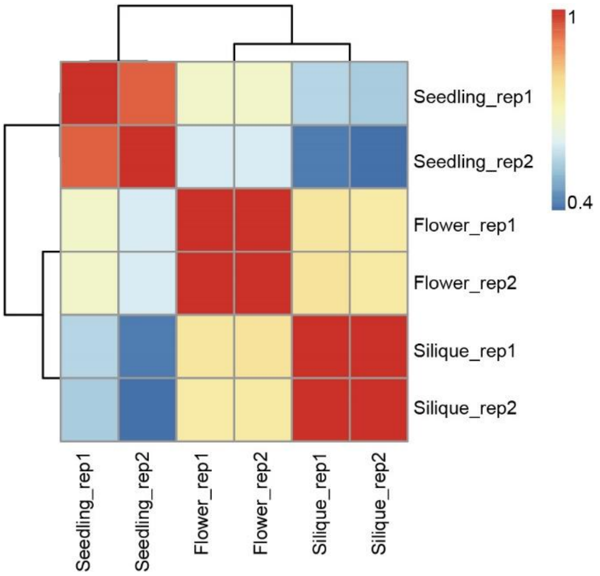

Supplementary Figure 3. Pearson correlation coefficient of RNA-seq.

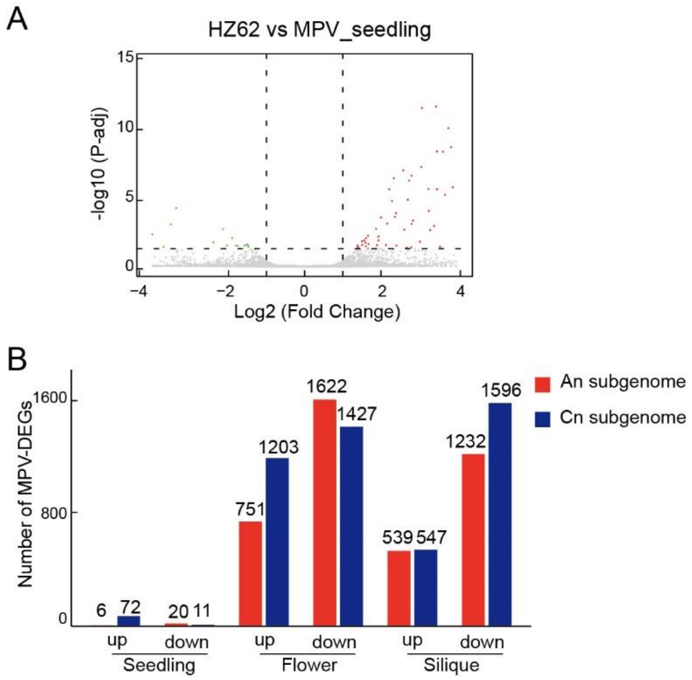

Supplementary Figure 4. MPV-DEGs in hybrid compared with parents. **(A)** Volcano plot of MPV-DEGs in hybrid seedling tissue. **(B)** Distribution of MPV-DEGs across the An and Cn subgenomes for all three tissues types.

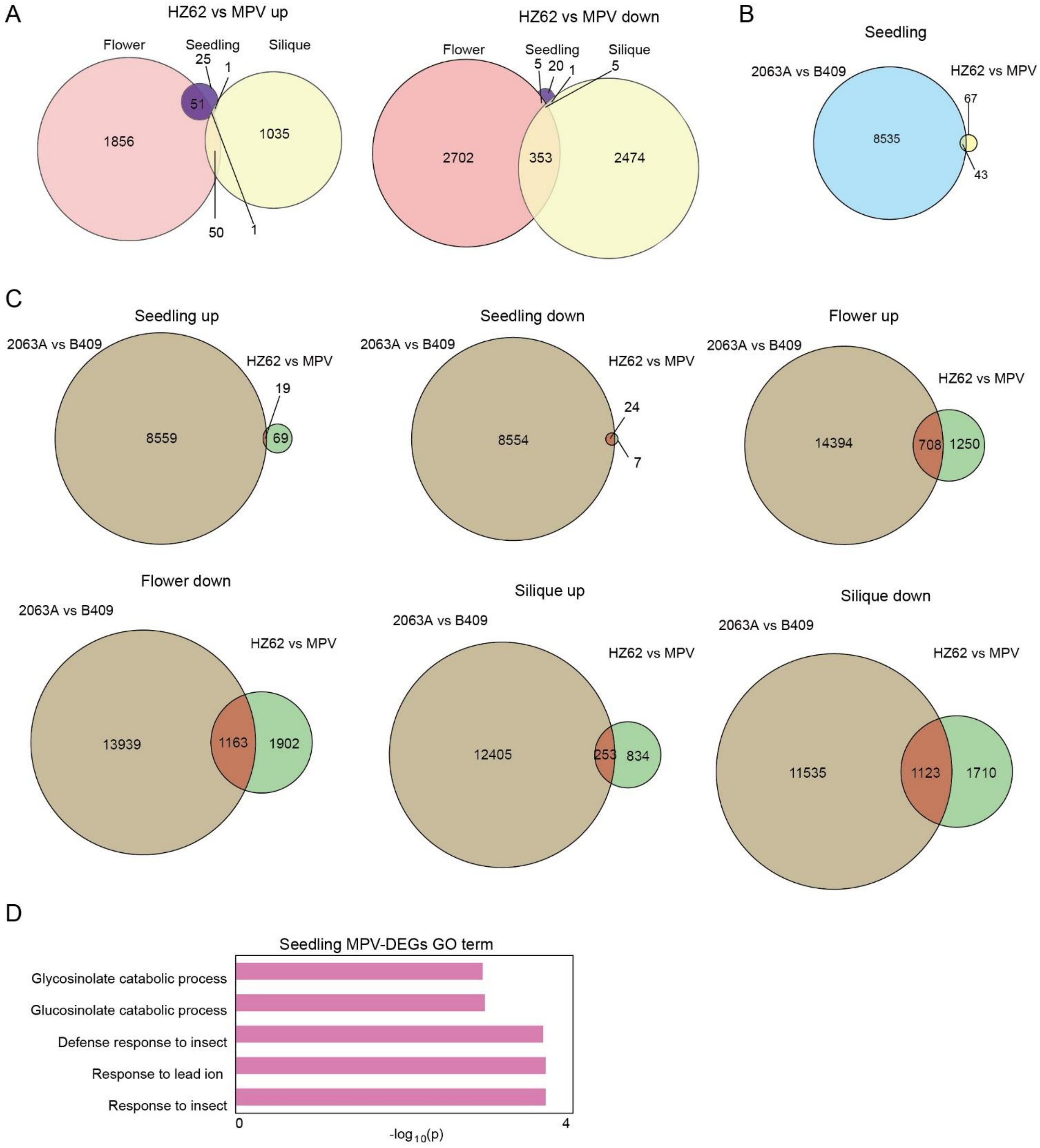

Supplementary Figure 5. MPV-DEGs in the hybrid. **(A)** Overlap of MPV-DEGs among three tissues. **(B)** Overlap of MPV-DEGs with parental DEGs in the indicated tissues. **(C)** Overlap of up-regulated and down-regulated MPV-DEGs with parental DEGs in seedling, flower bud, and silique tissues. **(D)** GO terms of MPV-DEGs in the silique.

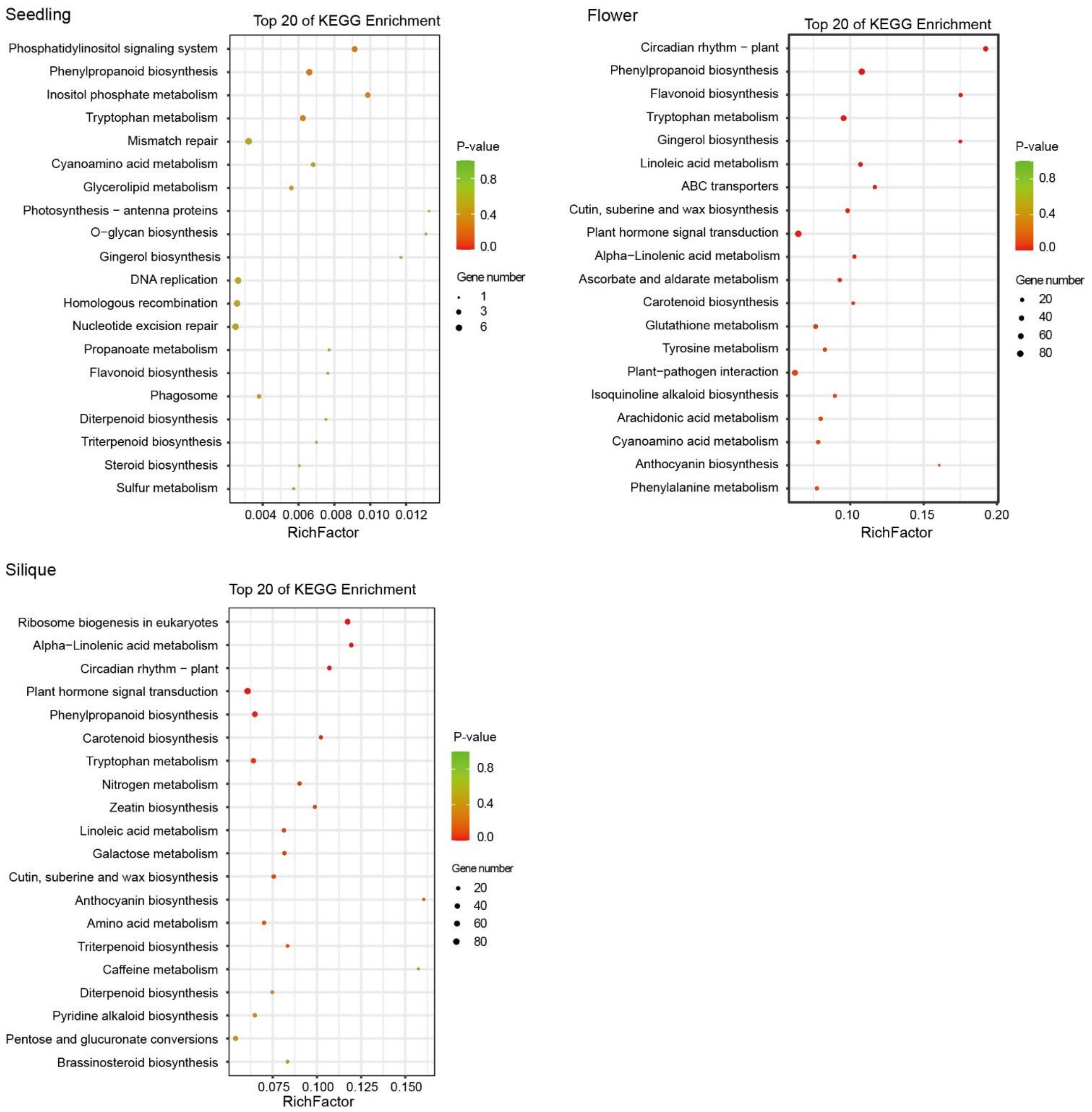

Supplementary Figure 6. KEGG analysis of MPV-DEGs in the indicated tissues.

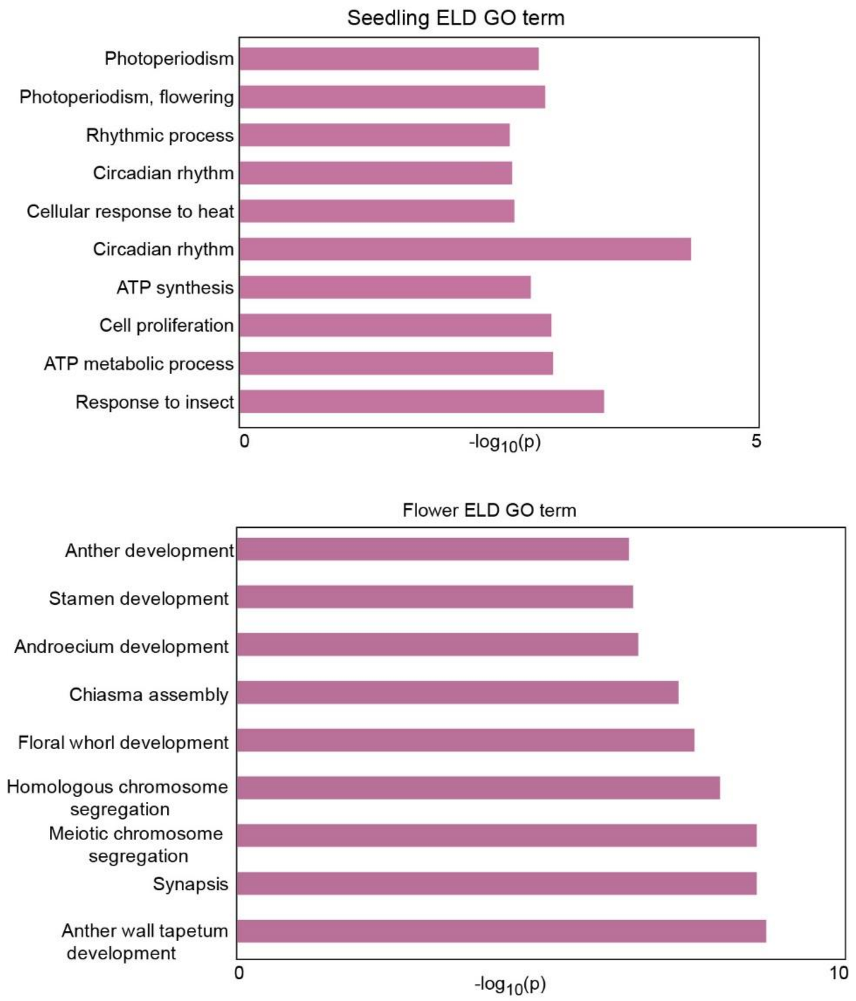

Supplementary Figure 7. GO analysis of ELD genes in seedling and flower bud.

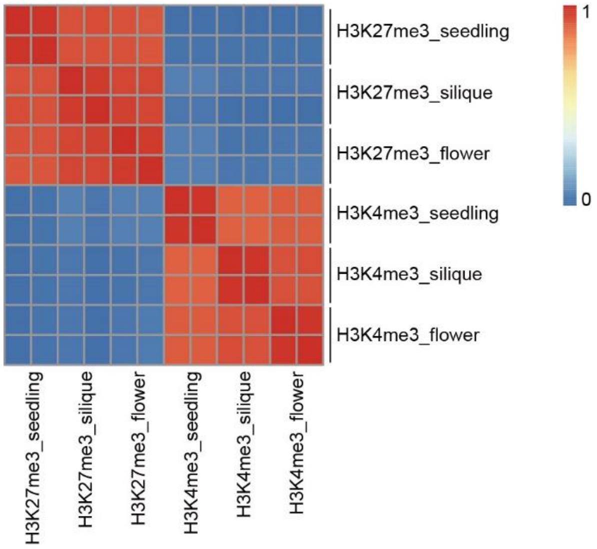

Supplementary Figure 8. Pearson correlation coefficient of ChIP-seq data.

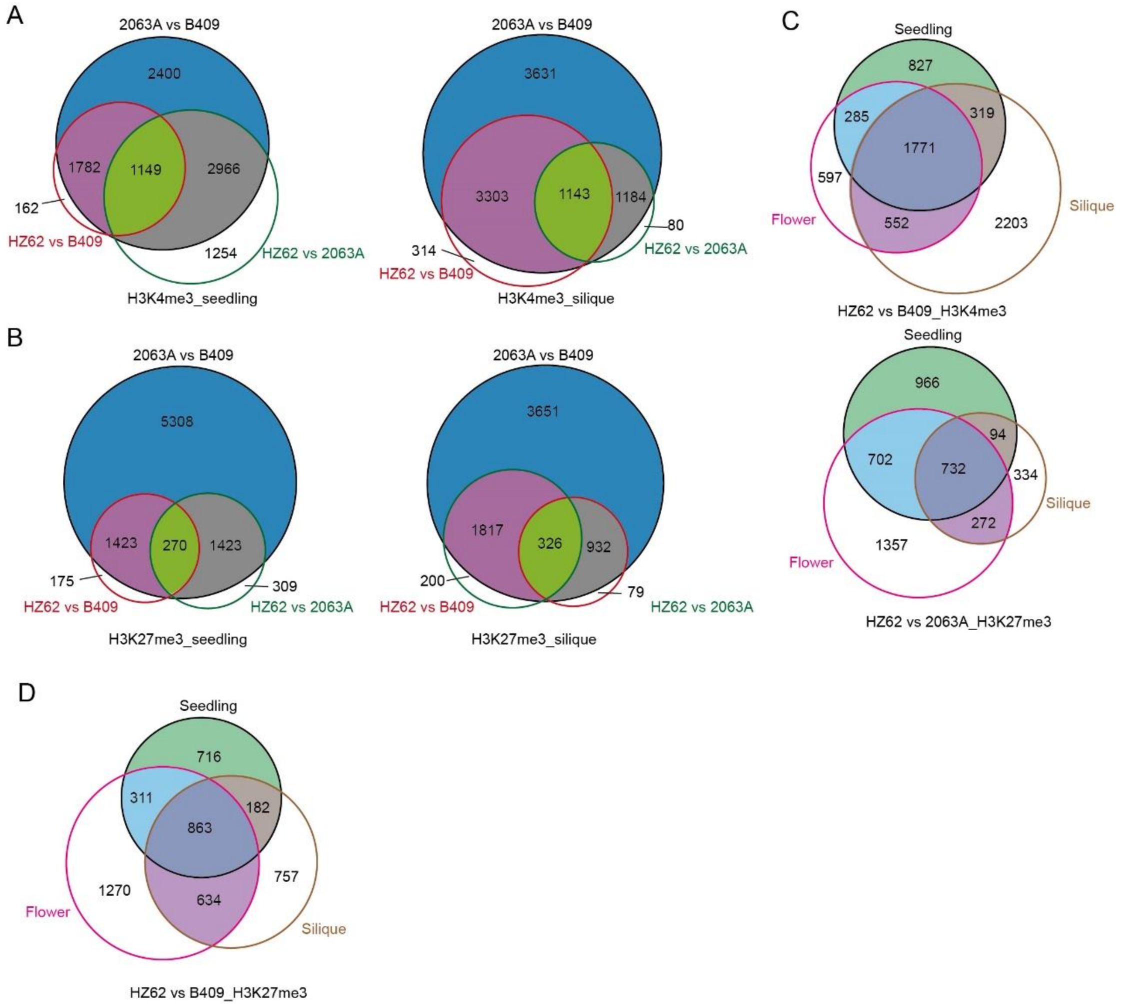

Supplementary Figure 9. Epigenetic variations in hybrid. **(A)** Venn diagram displaying the overlap of variation of H3K4me3 among parents and hybrid in the indicated tissues. **(B)** Overlap of variation of H3K27me3 among parents and hybrid in the indicated tissues. **(C)** Venn diagram displaying the overlap of variation of histone modifications between parents and hybrid for three tissue types.

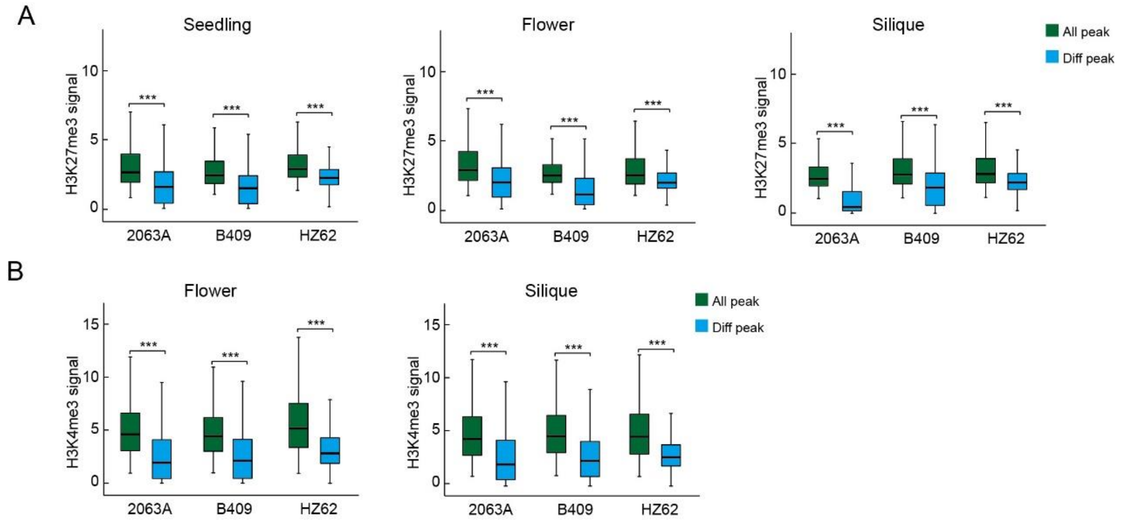

Supplementary Figure 10. The intensities of histone modifications in stable and variable regions. **(A)** Intensities of all and differential H3K4me3 peaks in the indicated varieties. **(B)** Intensities of all and differential H3K4me3 peaks in the indicated varieties. The statistical analysis wad performed using the Wilcoxon test. ****p* < 0.001.

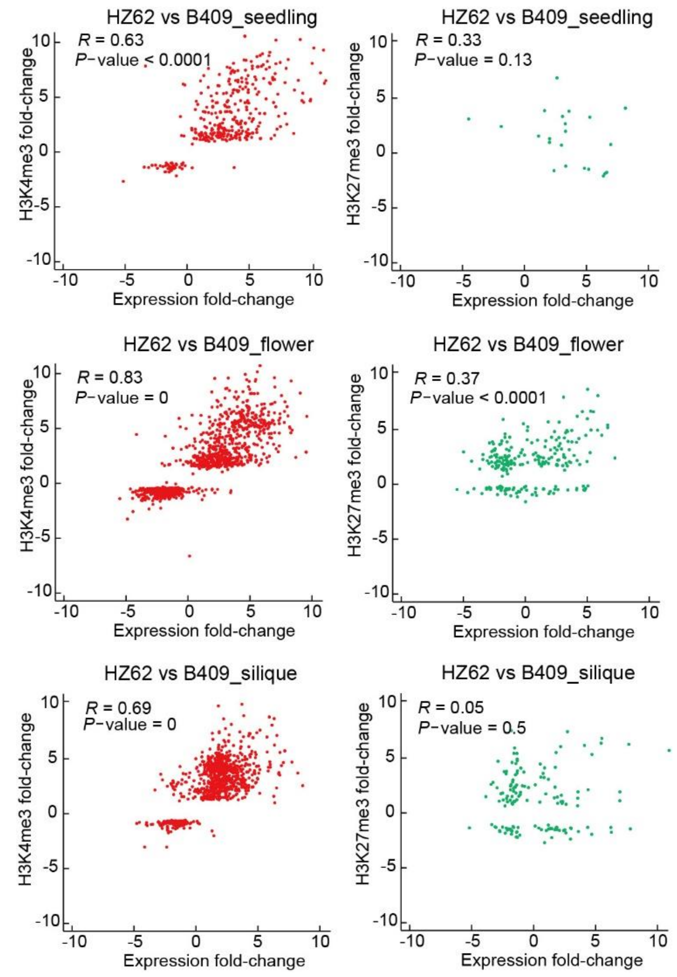

Supplementary Figure 11. Correlations between differential histone modification and gene expression. The statistical analysis was performed using the t-test.

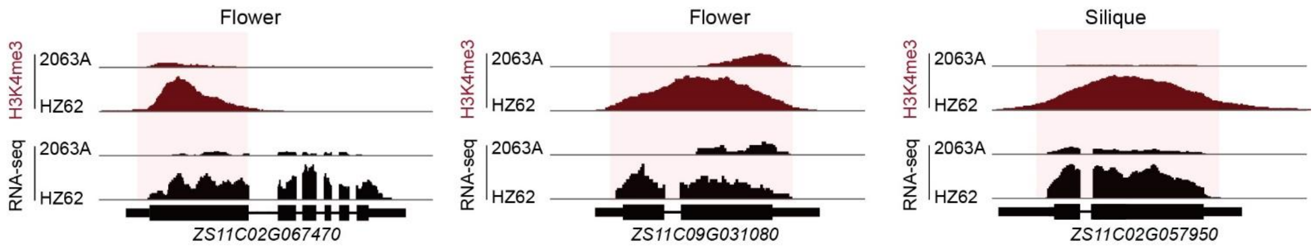

Supplementary Figure 12. Genome browsers of differentially H3K4me3-modified regions and expressed genes in HZ62 and 2063A.

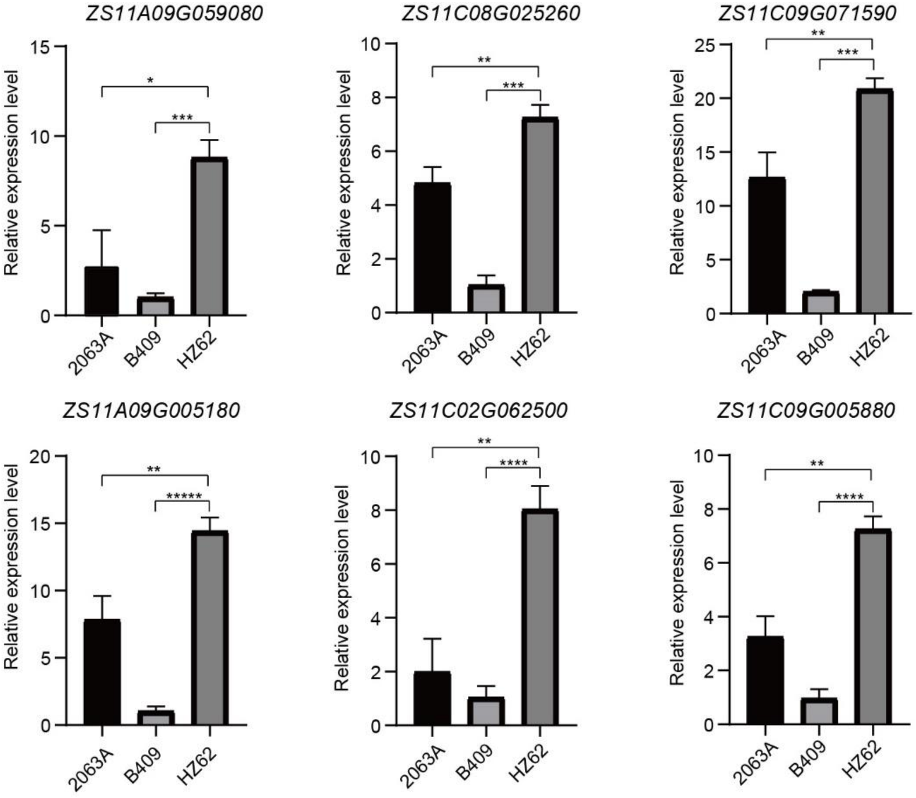

Supplementary Figure 13. Relative expression of starch metabolism related genes in parents and hybrid (*$p < 0.05$, ****$p < 0.0001$; t-test).

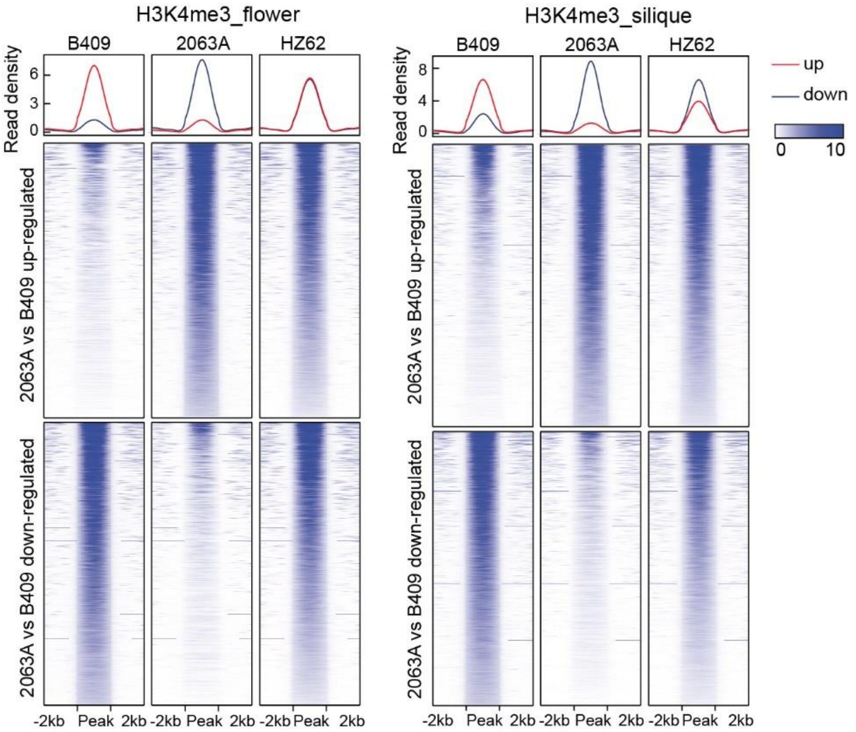

Supplementary Figure 14. Comparison of intensities of H3K4me3 in differentially histone-modified regions among parents and hybrid.

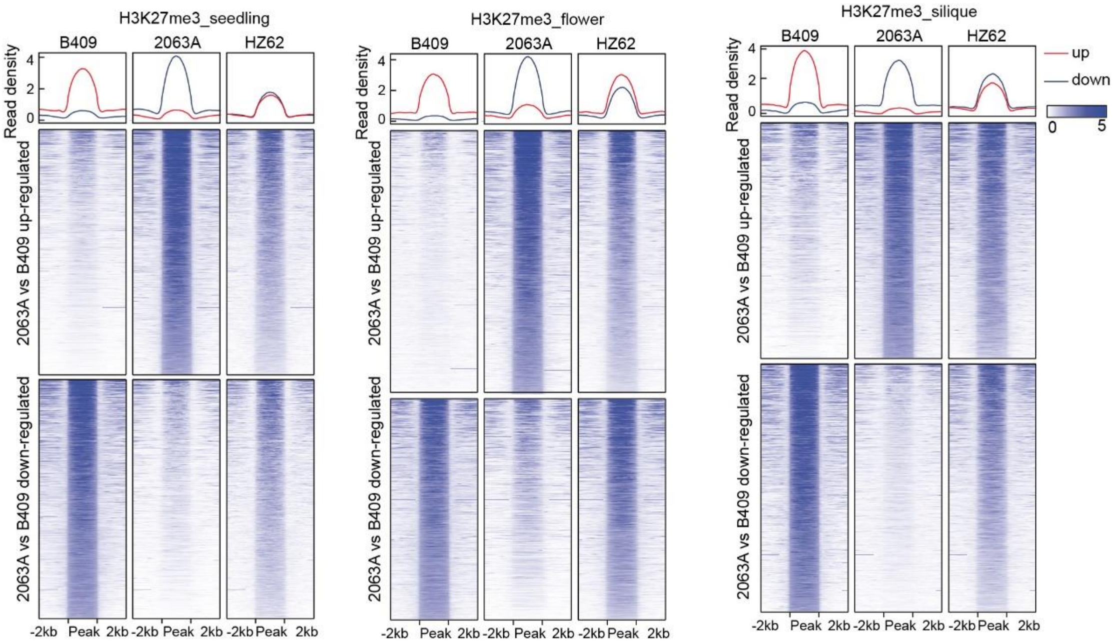

Supplementary Figure 15. Comparison of intensities of H3K27me3 in differentially histone-modified regions among parents and hybrid.

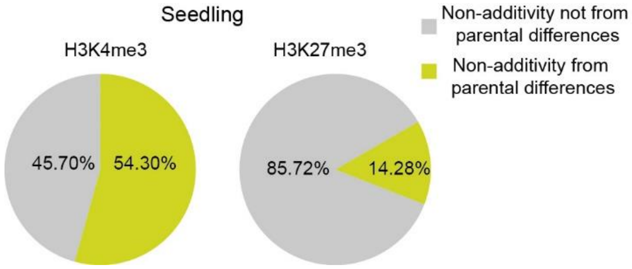

Supplementary Figure 16. The percentage of non-additively modified regions in hybrid originating from parental differentially histone-modified regions in the seedling.
